# Associations between prenatal phthalate exposure and maternal immune cell profiles across trimesters

**DOI:** 10.3389/fpubh.2026.1912015

**Published:** 2026-09-01

**Authors:** Yi Zhang, Lin Tao

**Affiliations:** 1 The People’s Hospital of Qian Nan Prefecture, Qian Nan, Guizhou, China; 2 Centre for Disease Control and Prevention, Huaxi District, Guiyang, Guizhou, China

**Keywords:** endocrine-disrupting chemicals, maternal immune cells, phthalates, pregnant women, trimester specific

## Abstract

**Introduction:**

Phthalates (PAEs) are endocrine-disrupting chemicals, yet the association between prenatal PAE exposure and maternal blood immune cells remains unclear. Here, we enrolled 2,581 pregnant women, including 287 in the first trimester (gestational weeks 1–12), 807 in the second trimester (gestational weeks 13–27), and 1,487 in the third trimester (gestational weeks 28 to delivery). This study aimed to elucidate the association between urinary phthalates (PAEs) metabolites during pregnancy and characteristics of the maternal immune cell profile.

**Methods:**

First, we analyzed differences in maternal PAE metabolites and immune cell counts (leukocytes, lymphocytes, neutrophils, eosinophils, basophils, monocytes, and platelets) in the three gestational stages using non-parametric alternatives (Kruskal-Wallis test). Subsequently, linear regression models quantified correlations between PAE metabolites and immune cell counts, while restricted cubic spline (RCS) analysis explored dose–response relationships between the two. Finally, Bayesian kernel machine regression (BKMR) was used to assess the non-linear associated between PAE metabolites and immune cells.

**Results:**

Findings revealed pregnancy-stage differences in both maternal PAE metabolites and maternal immune cell counts within each trimester. Linear regression identified significant associations between specific PAE metabolites and immune cell subsets, while RCS models further confirmed dose–response relationships for multiple metabolites. BKMR analysis highlighted a nonlinear association between combined exposure to PAE metabolites and basophils, monocytes, and lymphocytes.

**Discussion:**

In summary, this study has preliminarily established an association between prenatal maternal exposure to PAE metabolites and immune cells in maternal blood, highlighting the potential immunomodulatory effects of PAE exposure during pregnancy. However, as this study is primarily based on epidemiological research, all findings should be interpreted with caution.

## 1 Introduction

Phthalates (PAEs) are ubiquitous endocrine-disrupting chemicals (EDCs) with established carcinogenic and hormonal interference capacities (1). Despite the widespread acknowledgement of PAEs as hazardous EDCs, current scholarly efforts concerning their immunomodulatory potentials are still insufficient (2). Predominantly present in cosmetic products and plastic additives, PAEs invade human organisms through respiratory inhalation, oral intake and cutaneous penetration. These substances are ultimately eliminated via urine and feces, hence urinary and blood-borne PAE metabolites serve as reliable biomarkers for evaluating internal PAE exposure burdens (3). Following internal absorption, PAE contaminants and their metabolic derivatives accumulate in blood, urine and breast milk. Owing to their capability to penetrate the placental barrier, these compounds may interfere with fetal reproductive development (4–7). Emerging epidemiological data further verify the correlation between PAE contamination and adverse maternal health outcomes (8). Prior toxicological studies have validated that PAEs trigger endocrine disruption, immunotoxicity, cytotoxicity, genotoxicity and reproductive toxicity (9–11); nevertheless, human-based research focusing on PAE-induced immunotoxicity still remains underexplored.

The mammalian immune system constitutes an intricate and finely regulated biological network, and its functional dysregulation is closely associated with the occurrence of multiple non-communicable disorders (12). Extrinsic environmental contaminants, whether artificially synthesized or naturally occurring, have been proven to facilitate the progression of immune dysfunctions (13, 14). Pregnancy, especially the first trimester, is characterized by drastic hormonal alterations that restructure the maternal immune system. Such physiological changes render pregnant women more vulnerable to gestational complications and reduce bodily immunity. Maternal immune cells, such as natural killer (NK) cells, macrophages, T lymphocytes and dendritic cells, are indispensable for successful pregnancy maintenance. These immune subsets not only prevent fetal allograft rejection but also facilitate trophoblast infiltration, placental vascularization and gestational tissue remodeling (15–17).

Derived from hematopoietic stem cells that undergo maturation in the fetal liver and bone marrow (18, 19), the above immune cells participate in both humoral and cellular immune responses. Even with low abundance in circulation, these immune populations can synthesize antibodies and regulatory bioactive molecules, and their relevance to maternal-fetal wellness has attracted escalating research interest (20). Immune cell profiles undergo physiological changes throughout gestation: leukocyte and neutrophil levels moderately rise between the 27th and 33rd gestational weeks before declining in late pregnancy, while eosinophil and basophil counts present a sustained downward trend from early gestation to full-term delivery (21–23).

Accordingly, the present study enrolled 2,581 pregnant females from the Zunyi Birth Cohort and adopted a multi-method statistical analytical framework. This research aimed to systematically explore trimester-specific correlations between urinary PAE metabolite concentrations and maternal peripheral blood immune cell quantities. The obtained epidemiological evidence can provide actionable references for targeted maternal and child health intervention strategies.

## 2 Methods

### 2.1 Study population

The study population was drawn from the Zunyi Birth Cohort (ZBC), is a prospective study established between April 2020 and May 2022, aiming to investigate the associated with environmental and behavioral factors on fetal development and pregnancy outcomes (24, 25). For this study, we enrolled 2,581 pregnant women, including 287 in the first trimester (gestational weeks 1–12), 807 in the second trimester (gestational weeks 13–27), and 1,487 in the third trimester (gestational weeks 28 to delivery). All participants contributed complete, analyzable data, including baseline information, urinary PAE exposure levels, and maternal immune cell markers. See Supplementary Figure S1.

### 2.2 Sample collection and testing

In the present study, urinary PAE metabolites were analyzed using gas chromatography–mass spectrometry (GC–MS; Agilent 9,000-7000D system, Agilent Technologies, Inc., Santa Clara, CA, United States). Detailed protocols for the specific assay are described in previous studies. The limits of detection (LOD), limits of quantification (LOQ), recoveries, and precision for all PAE metabolites analyzed in this study are presented in Supplementary Tables S1, S2 (24, 25).

### 2.3 Research variables

The independent variables in this study were obtained from laboratory data, dependent variables from the hospital system, and basic information about the pregnant women and their families were obtained from responses to a questionnaire. The independent variables included monoethyl phthalate (MEP), monobutyl phthalate (MBP), monobutyl phthalate (MiBP) and monomethyl phthalate (MMP), monobutyl phthalate (MnBP), mono(2-ethylhexyl) phthalate (MEHP), mono(2-ethyl-5-hydroxyhexyl) phthalate (MEHHP), mono(2-ethyl-5-oxohexyl) phthalate (MEOHP), mono(2-ethyl-5) octyl phthalate (MOP), monophenyl phthalate (MBzP). The dependent variables included maternal leukocytes (×10^9^/L), lymphocytes (×10^9^/L), neutrophils (×10^9^/L), eosinophils (×10^9^/L), basophils (×10^9^/L), monocytes (×10^9^/L), and platelets (×10^9^/L). Adjustment factors were primarily selected based on three key categories: PAE exposure sources (tobacco smoke and occupational exposure), physiological susceptibility factors [maternal age and pre-pregnancy body mass index (BMI)], and socioeconomic determinants (educational attainment and household income). These variables have been previously reported to independently modulate immune cell function or interact with PAE exposure, thereby potentially confounding the estimated associations between urinary PAE metabolites and circulating immune profiles. Fully adjusted covariates included maternal age, maternal educational attainment, maternal occupation, marital status, pre-pregnancy BMI, maternal smoking status, partner’s educational attainment, partner’s occupation, annual household income, gestational week at sampling, and partner’s smoking history.

### 2.4 Definition and quantitative analysis of immune cells

The immunological parameters included in this study comprise: leukocyte count (×10^9^/L), lymphocyte count (×10^9^/L), neutrophil count (×10^9^/L), eosinophil count (×10^9^/L), basophil count (×10^9^/L), monocyte count (×10^9^/L), and platelet count (PLT, ×10^9^/L). All parameters were defined according to the Basic Principles and Methods for Interpretation of Clinical Laboratory Results (WS/T 402–2012) and the Guidelines for Approval of Blood Analyzers and Related Tests (H20-A2). To ensure accuracy and reproducibility, a dual-quantification strategy was employed, combining routine clinical testing with internal laboratory validation. The specific workflow is as follows: Firstly, blood samples were collected from pregnant female subjects in a fasting, resting state, avoiding periods of uterine contraction. Vacuum blood collection tubes containing EDTA-K2 (1.8 mg EDTA-K2 per milliliter of blood) were used. Samples must undergo testing within 1 h of collection. Should delayed testing be necessary, samples were stored at 4 °C (maximum 4 h), followed by equilibration at room temperature for 30 min before analysis. Secondly, the XN-9000 fully automated blood analyser, manufactured by Sysmex Corporation of Japan, was employed. This instrument utilizes a combined ‘electrical impedance + laser scattering’ detection technology to simultaneously perform white blood cell counts (including differential counts: neutrophils, lymphocytes, etc.) and platelet counts. Finally, manual verification is conducted using Wright-Giemsa staining. Under oil immersion microscopy, 200 white blood cells are counted to calculate differential percentages. These percentages are compared with analyser results, requiring >90% concordance (Kappa > 0.85). In case of discrepancy, manual results prevail. All results are synchronously entered into the Laboratory Information System (LIS), recording blood collection time, analysis time, instrument serial number, quality control batch number, and operator details. Data is linked to subject pregnancy information (gestational age, number of antenatal visits) to establish a complete traceability chain supporting subsequent data validation and repeatability verification.

### 2.5 Statistical analysis

This study adopted a cross-sectional design, with pregnant women in the first, second, and third trimesters serving as independent samples at distinct gestational weeks. Notably, these samples did not derive from the same cohort followed in all three trimesters. Data normality was assessed via the Kolmogorov–Smirnov test. Continuous variables were presented as mean ± standard deviation (SD), categorical variables as percentages, and non-normally distributed variables as median (interquartile range, IQR). Given the right-skewed distribution of phthalate ester (PAE) metabolite concentrations, natural logarithmic transformation was applied. Differences in maternal urinary PAE metabolite concentrations and immune cell levels in gestational stages were analyzed using non-parametric alternatives (Kruskal-Wallis test). Correlations between PAE metabolite levels and immune cell counts in gestational stages were assessed via linear regression models. Exposure–response relationships between PAE metabolite levels and immune cell counts within each trimester were explored using restricted cubic spline (RCS) plots. Finally, given the common co-occurrence of PAE metabolites in the environment, Bayesian kernel machine regression (BKMR) was employed to evaluate the association between mixed exposure to multiple PAE metabolites and immune cell lineages. All models were adjusted for potential confounders. Data analysis was performed using SPSS 29.0 and R 3.4.0 (obtained from the Comprehensive R Archive Network, CRAN). Statistical significance was defined as *p* < 0.05 (two-tailed test). Specific R packages used included BKMR and rms: the BKMR package was used to analyze associations between maternal PAE metabolite exposure and immune cell populations; restricted cubic spline (RCS) analysis—a regression modeling approach—was implemented via the rcs() function in the rms package.

## 3 Results

### 3.1 Characteristics of participants

This study included 2,581 pregnant women, with 287 in the first, 807 in the second, and 1,487 in the third trimester. Gestational ages for each trimester were: first trimester: 10 ± 2 weeks [mean ± standard deviation (SD)]; second trimester: 21 ± 4 weeks (mean ± SD); third trimester: 36 ± 3 weeks (mean ± SD). Demographically, participants were predominantly aged 20–35 years (94%); 80% of household heads had a secondary education, and 95% of participants were married. Occupational status was homogeneous within each trimester (60%). Active smoking during pregnancy was rare, whereas frequent second-hand smoke exposure was commonly reported. Differential analyses identified significant differences in the three trimesters in maternal occupational history, pre-pregnancy body mass index (BMI), spousal occupational history, and annual household income. Detailed baseline characteristics for each trimester are provided in Table 1.

**Table 1:** Baseline information about pregnant women.

Variable	The first trimester (*N* = 287)	The second trimester (*N* = 807)	The third trimester (*N* = 1,487)	*χ* ^2^	*p*
Age (years)					
20–35	270 (94.07%)	760 (94.18%)	1,396 (93.88%)	0.085	0.958
36–45	17 (5.93%)	47 (5.82%)	91 (6.12%)		
Education					
Low	8 (2.78%)	26 (3.22%)	52 (3.50%)	4.628	0.328
Middle	228 (79.44%)	631 (78.19%)	1,206 (81.10%)		
High	51 (17.78%)	150 (18.59%)	229 (15.40%)		
Employment status					
Employed	190 (66.20%)	507 (62.83%)	798 (53.67%)	27.09	**<0.001**
Unemployed	97 (33.80%)	300 (37.17%)	689 (46.33%)		
Marriage status					
Married	276 (96.17%)	767 (95.04%)	1,417 (95.29%)	0.601	0.740
Unmarried	11 (3.83%)	40 (4.96%)	70 (4.71%)		
Pre-pregnancy BMI (kg/m^2^)					
<18.5	36 (12.54%)	91 (11.28%)	165 (11.10%)	43.409	**<0.001**
18.5–23.9	165 (57.49%)	504 (62.45%)	922 (62.00%)		
>23.9	86 (29.97%)	212 (26.27%)	400 (26.90%)		
Spouse’s education					
Low	2 (0.7%)	12 (1.49%)	23 (1.55%)	7.124	0.129
Middle	222 (77.35%)	638 (79.06%)	1,217 (81.84%)		
High	63 (21,95%)	157 (19.45%)	247 (16.61%)		
Spouse’s employment status					
Employed	250 (87.11%)	679 (84.14%)	1,123 (75.52%)	35.298	**<0.001**
Unemployed	37 (12.89%)	128 (15.86%)	364 (24.48%)		
Annual household income(yuan)					
<100,000	56 (19.51%)	150 (18.59%)	578 (38.87%)	121.397	**<0.001**
100,000–200,000	199 (69.34%)	584 (72.37%)	807 (54.27%)		
>200,000	32 (11.15%)	73 (9.04%)	102 (6.86%)		
Active smoking					
Yes	3 (1.05%)	13 (1.61%)	13 (0.87%)	3.091	0.213
No	284 (98.95%)	794 (98.39%)	1,474 (99.13%)		
Spouse’s smoking					
Yes	102 (35.54%)	307 (38.04%)	551 (37.05%)	0.597	0.742
No	185 (64.46%)	500 (61.96%)	936 (62.95%)		
Gestation week					
NA	10.72 ± 2.02	21.17 ± 4.66	36.25 ± 3.60	NA	NA

Low: primary school education and below. Middle: junior high school education. High: college education and beyond. Bold values indicate statistically significant differences.

### 3.2 Concentrations of PAE metabolites in the urine and levels of immune cells in the blood of pregnant women

**Figure 1:**
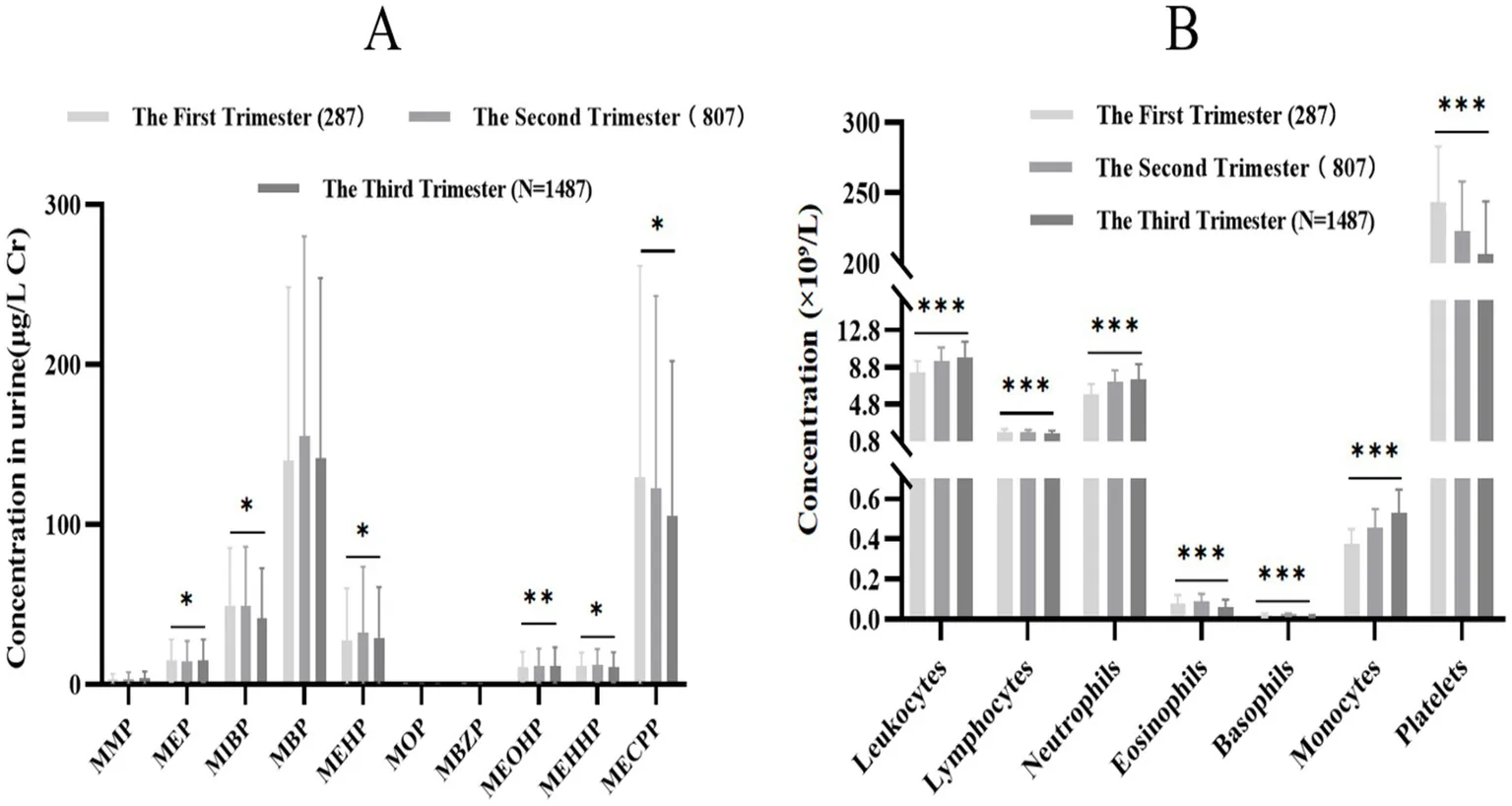
Maternal urinary concentrations of PAE metabolites and blood levels of immune cells. **(A)** Concentration of PAE metabolites in the urine of pregnant women during pregnancy (µ g/L Cr), **(B)** Count of immune cells in the blood of pregnant women during pregnancy.

Given the non-normal distribution of PAE metabolite concentrations and immune cell counts, these variables are presented as median values (95% confidence interval, CI). Within the study cohort, 94 pregnant women exhibited phthalate monobutyl ester (MBP) concentrations below the limit of detection (17 in first Trimester, 29 in second Trimester, 48 in third Trimester), while 74 pregnant women had phthalate monoethyl ester (MEP) concentrations below the limit of detection (9 in first Trimester, 23 in second Trimester, 42 in third Trimester). 11 pregnant women had monoisobutyl phthalate (MIBP) concentrations below the limit of detection (0 in first Trimester, 2 in second Trimester, 9 in third Trimester), 54 pregnant women had mono-n-octyl phthalate (MOP) concentrations below the limit of detection (12 in first Trimester, 18 in second Trimester, 24 in third Trimester), 49 cases had mono(2-ethylhexyl) phthalate (MEHP) concentrations below the detection limit (8 in first Trimester, 17 in second Trimester, 24 in third Trimester), 25 cases of mono(2-ethyl-5-oxohexyl) phthalate (MEOHP) were below the detection limit (2 in first Trimester, 9 in second Trimester, 14 in third Trimester), 68 cases of mono-benzyl phthalate (MBZP) were below the detection limit (13 in first Trimester, 19 in second Trimester, 36 in third Trimester), 96 samples had phthalate mono(2-ethyl-5-hydroxyhexyl) ester (MEHHP) concentrations below the detection limit (13 in first Trimester, 26 in second Trimester, 57 in third Trimester), and 62 samples had phthalate mono(2-ethyl-5-carboxyhexyl) ester (MECPP) concentrations below the detection limit (21 in first Trimester, 15 in second Trimester, 26 in third Trimester). Detection limits for PAE metabolites are provided in Supplementary Table S2. Next, during the first trimester, MBP concentrations were the highest at 113.4 (47.4, 259.3) μg/L creatinine (Cr), whereas MBZP concentrations were the lowest at 0.096 (0.046, 0.336) μg/L Cr. In the second trimester, MBP concentrations were highest at 124.3 (48.1–292.6) μg/L Cr, while MBZP remained the least abundant at 0.094 (0.038–0.274) μg/L Cr. In the third trimester, MBP concentrations were highest at 112.6 (45.1–265.6) μg/L Cr, and MBZP concentrations remained the lowest at 0.150 (0.068–0.371) μg/L Cr (see Supplementary Table S3). Furthermore, Kruskal-Wallis test revealed significant differences in maternal plasma concentrations of MEP, MIBP, MEHP, MEOHP, MEHHP, and MECPP within each trimester. Additionally, this analysis uncovered a distinct variation in maternal immune cell counts: white blood cells, neutrophils, and monocytes showed sustained increases within each trimester, while lymphocytes and platelets decreased progressively. Eosinophil and basophil counts increased initially followed by a decline (Figure 1). Pearson correlation analysis revealed that the seven substances—MEP, MIBP, MBP, MEHP, MOP, MEOHP, and MEHHP—exhibited very strong to strong positive correlations (with correlation coefficients ranging mostly between 0.76 and 0.98). Among these, the correlation coefficients between MEP and both MEOHP and MEHHP were as high as 0.98, indicating the most significant correlations; In contrast, MMP, MBZP, and MECPP showed weaker correlations with most of the other compounds (see Supplementary Figure S2).

### 3.3 Association of PAE metabolites with maternal immune cells

**Figure 2:**
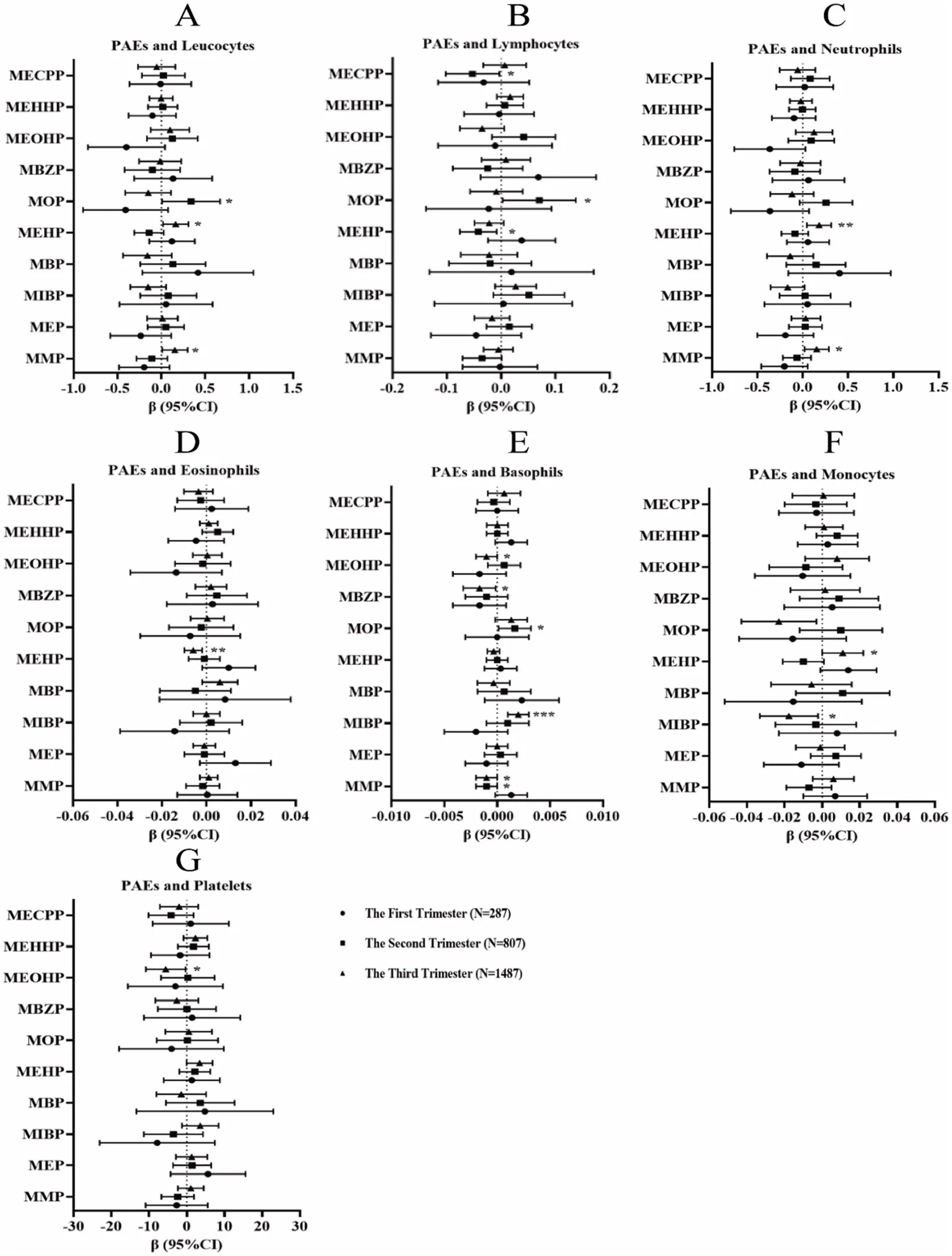
Association of PAE metabolites with maternal immune cells. Adjustment factors: maternal age, maternal educational attainment, maternal occupation, maternal marital status, pre-pregnancy BMI, maternal smoking history, spousal educational attainment, spousal occupation, annual household income, gestational age and spousal smoking history. **(A)** Association between PAE metabolites and leukocytes, **(B)** Association between PAE metabolites and lymphocytes, **(C)** Association between PAE metabolites and neutrophils, **(D)** Association between PAE metabolites and eosinophils, **(E)** Association between PAE metabolites and basophils, **(F)** Association between PAE metabolites and monocytes, **(G)** Association between PAE metabolites and platelets.

Linear regression models evaluated associations between PAE metabolites and maternal immune cell levels within each trimester. No significant associations were observed in the first trimester. In the second trimester, MOP was positively associated with leukocytes (*β* = 0.34, 95% CI: 0.01–0.67), while MEHP, MOP, and MECPP showed associations with lymphocytes (*β* = −0.04, 95% CI: −0.08 to −0.01; *β* = 0.07, 95% CI: 0.003–0.12; *β* = −0.05, 95% CI: −0.10 to −0.003, respectively). MMP and MOP were linked to basophils (*β* = −0.001, 95% CI: −0.002 to 0.00; *β* = 0.002, 95% CI: 0.00–0.003, respectively). in the third trimester, MMP and MEHP were positively associated with leukocytes (*β* = 0.16, 95% CI: 0.01–0.30; *β* = 0.16, 95% CI: 0.02–0.31, respectively), while MEP and MEHP correlated with neutrophils (*β* = 0.15, 95% CI: 0.02–0.29; *β* = 0.18, 95% CI: 0.05–0.31, respectively). MEHP was negatively associated with eosinophils (*β* = −0.006, 95% CI: −0.01 to −0.002). For basophils, MMP, MBZP, and MEOHP showed subtle associations (*β* = −0.001, 95% CI: −0.002 to 0.00; *β* = −0.002, 95% CI: −0.003 to 0.00; *β* = −0.001, 95% CI: −0.002 to 0.00, respectively), while MIBP and MOP were negatively linked to monocytes (*β* = −0.02, 95% CI: −0.03 to −0.002; *β* = −0.02, 95% CI: −0.04 to −0.003, respectively). MEOHP was associated with reduced platelets (*β* = −5.58, 95% CI: −10.82 to −0.33; Figure 2).

### 3.4 Non-linear relationship between PAE metabolites and maternal immune cells

**Figure 3:**
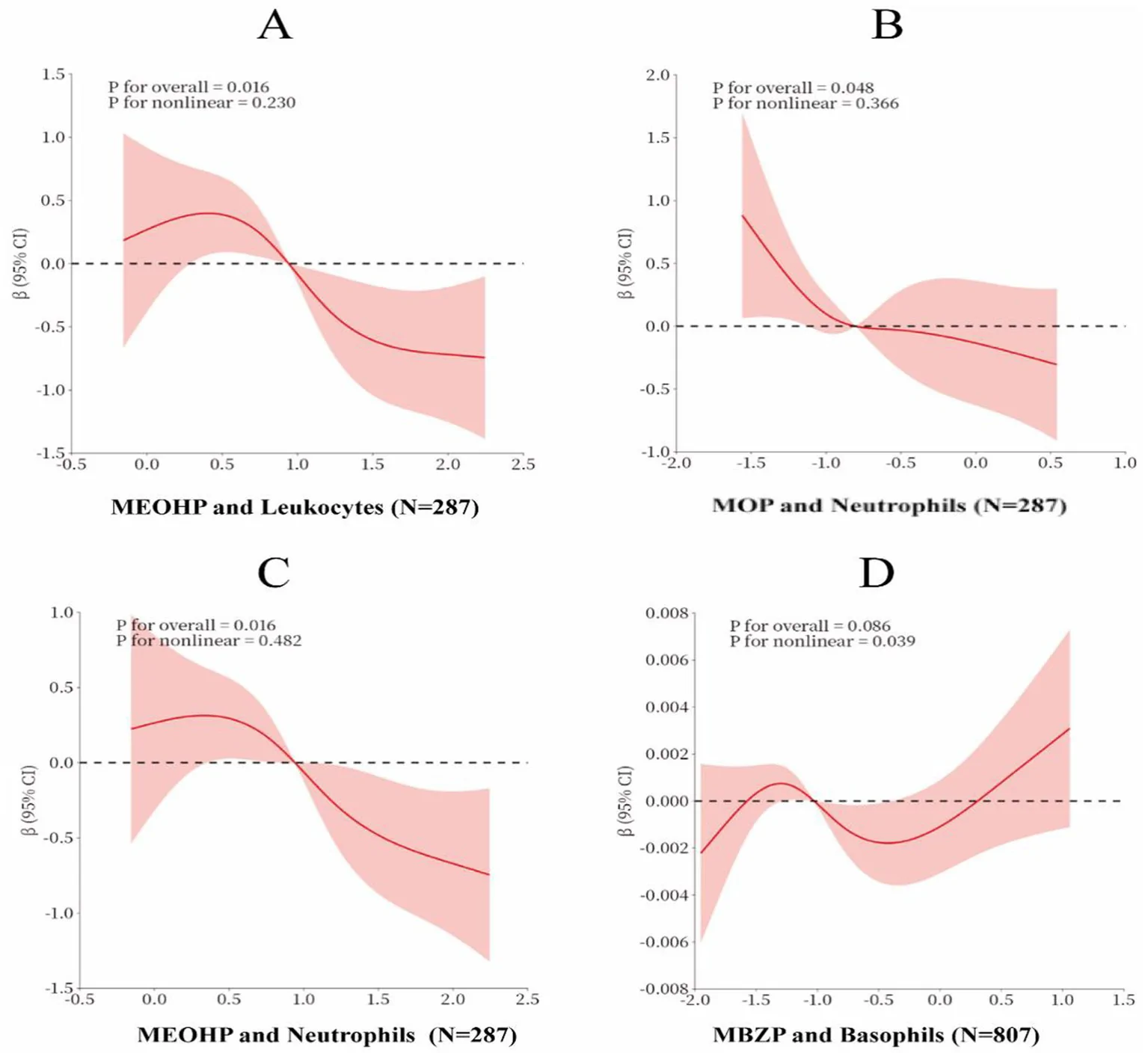
Non-linear relationship between metabolites of PAE metabolites s and maternal immune cells in the first and second trimester. **(A–C)** Show the non-linear relationships between phthalate (PAE) metabolites and maternal immune cells in the first trimester, whereas **(D)** shows the non-linear relationship between PAE metabolites and maternal immune cells in the second trimester. Adjustment factors: Maternal age, maternal educational attainment, maternal occupation, maternal marital status, pre-pregnancy BMI, maternal smoking history, spousal educational attainment, spousal occupation, annual household income, gestational age and spousal smoking history.

**Figure 4:**
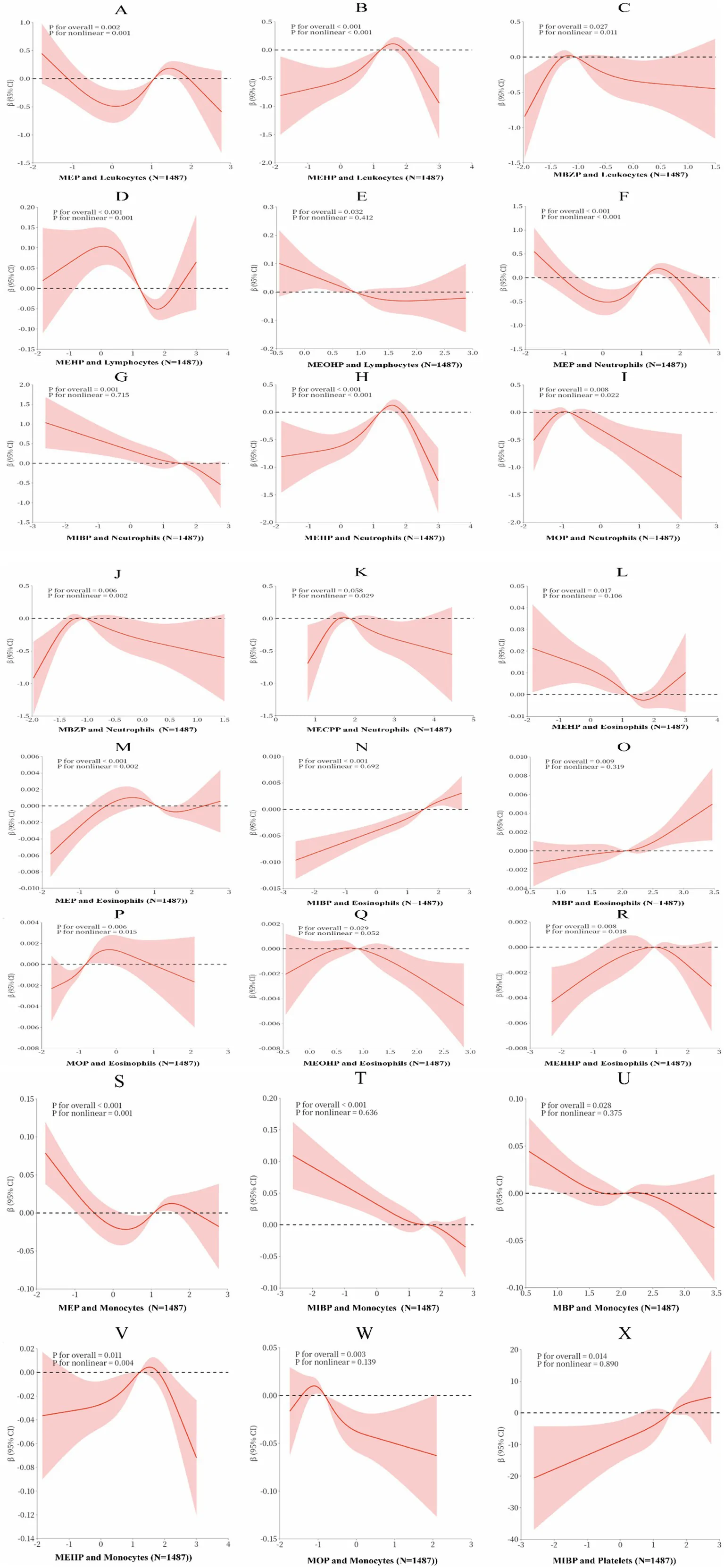
Non-linear relationship between PAE metabolites and maternal immune cell in the third trimester. Adjustment factors: maternal age, maternal educational attainment, maternal occupation, maternal marital status, pre-pregnancy BMI, maternal smoking history, spousal educational attainment, spousal occupation, annual household income, gestational age and spousal smoking history. **(A−E)** Nonlinear association between PAE metabolites and leukocytes, **(F−K)** Nonlinear association between PAE metabolites and neutrophils, **(L−R)** Nonlinear association between PAE metabolites and eosinophils, **(S−W)** Nonlinear association between PAE metabolites and monocytes, **(X)** Nonlinear association between PAE metabolites and platelets.

Restricted cubic spline curves were employed to further characterize the relationships between phthalate (PAE) metabolites and maternal immune cell counts. During the first trimester, non-linear associations were observed between MEOHP and leukocyte counts (overall *p* < 0.05), and between MOP or MEOHP and neutrophil/monocyte counts (overall *p* < 0.05; Figure 3). In the second trimester, MBZP showed a non-linear association with basophil counts (overall *p* < 0.05). During the third trimester, MEP, MEHP, and MBZP exhibited non-linear associations with leukocyte counts (overall *p* < 0.05), while MEHP and MEOHP showed non-linear associations with lymphocyte counts (overall p < 0.05). Multiple metabolites (MEP, MIBP, MEHP, MOP, MBZP, MECPP) also displayed non-linear associations with neutrophil counts (overall *p* < 0.05), with MEHP showing a specific non-linear association with eosinophil counts (overall *p* < 0.05). For basophils, MEP, MIBP, MBP, MOP, MEOHP, and MEHHP exhibited non-linear associations (overall *p* < 0.05), whereas monocytes showed non-linear associations with MEP, MIBP, MBP, MEHP, and MOP (overall *p* < 0.05). Additionally, MIBP showed a non-linear association with platelet counts (overall *p* < 0.05; Figure 4).

### 3.5 Combined association between PAE metabolites and maternal immune cells

**Figure 5:**
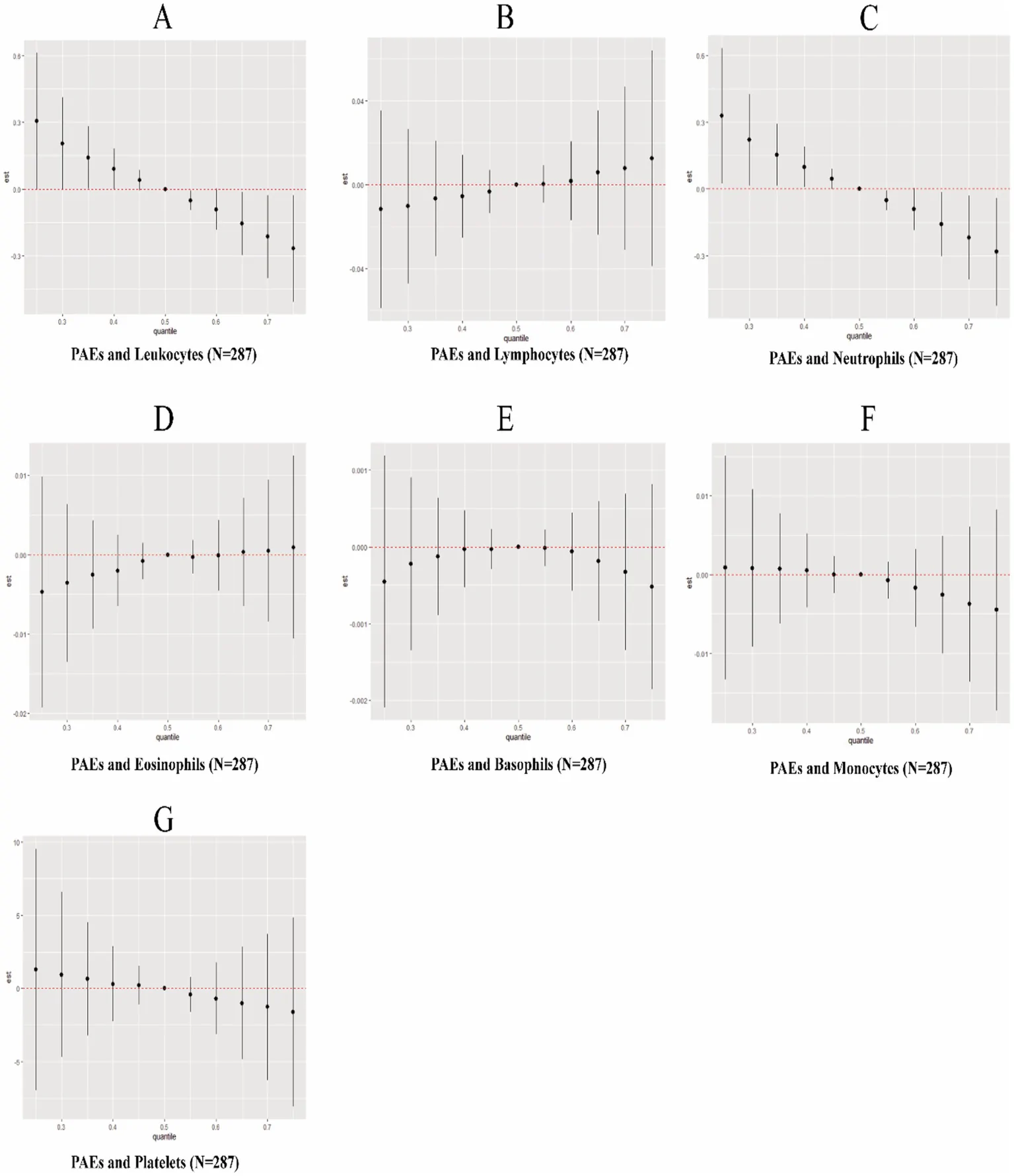
Combined associated between PAE metabolites and maternal immune cells in the first trimester. Adjustment factors: maternal age, maternal educational attainment, maternal occupation, maternal marital status, pre-pregnancy BMI, maternal smoking history, spousal educational attainment, spousal occupation, annual household income, gestational age and spousal smoking history. **(A)** Mixed association between PAE metabolites and leukocytes, **(B)** Mixed association between PAE metabolites and lymphocytes, **(C)** Mixed association between PAE metabolites and neutrophils, **(D)** Mixed association between PAE metabolites and eosinophils, **(E)** Mixed association between PAE metabolites and basophils, **(F)** Mixed association between PAE metabolites and monocytes, **(G)** Mixed association between PAE metabolites and platelets.

**Figure 6:**
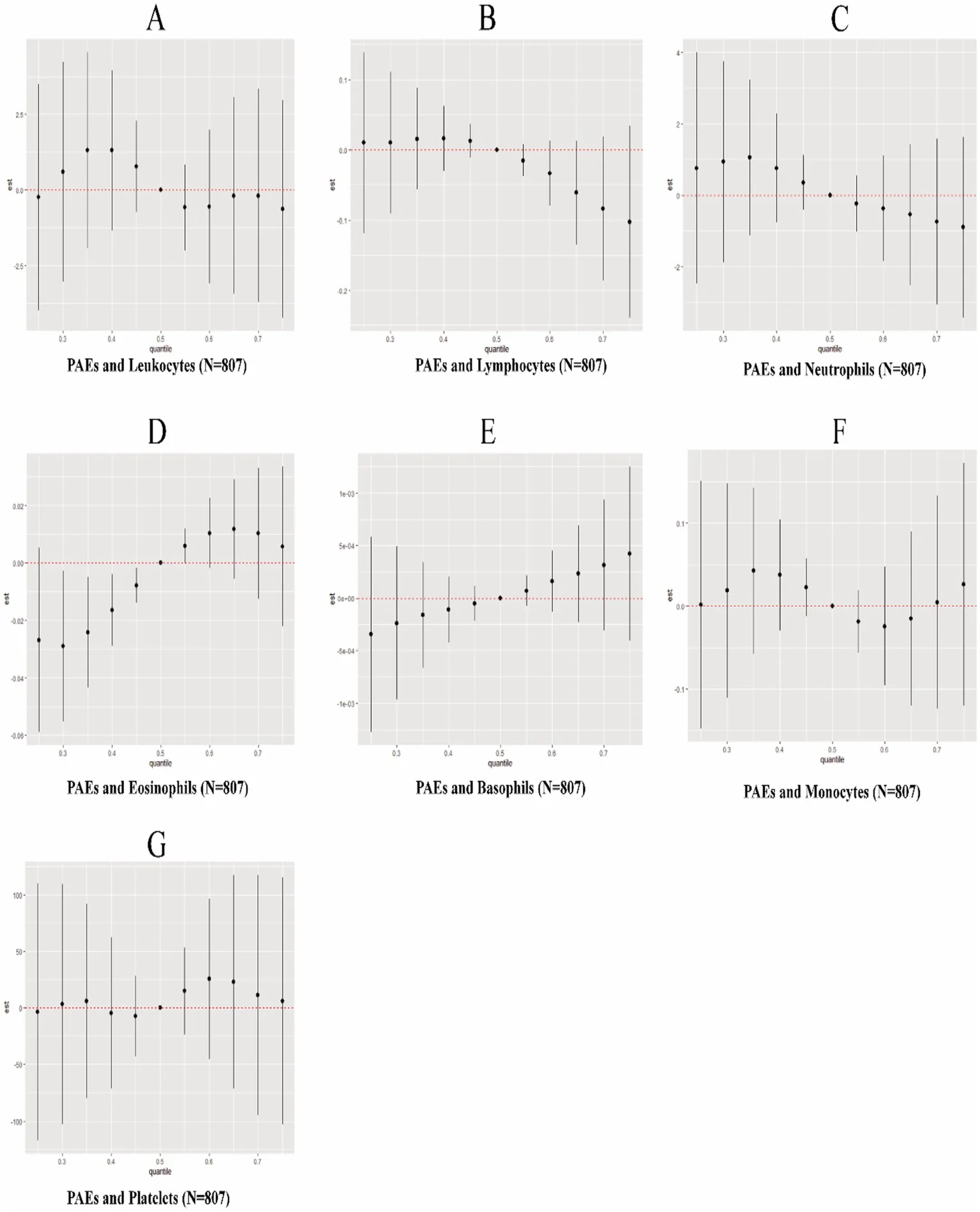
Combined associated between PAE metabolites and maternal immune cells in the second trimester. Adjustment factors: maternal age, maternal educational attainment, maternal occupation, maternal marital status, pre-pregnancy BMI, maternal smoking history, spousal educational attainment, spousal occupation, annual household income, gestational age and spousal smoking history. **(A)** Mixed association between PAE metabolites and leukocytes, **(B)** Mixed association between PAE metabolites and lymphocytes, **(C)** Mixed association between PAE metabolites and neutrophils, **(D)** Mixed association between PAE metabolites and eosinophils, **(E)** Mixed association between PAE metabolites and basophils, **(F)** Mixed association between PAE metabolites and monocytes, **(G)** Mixed association between PAE metabolites and platelets.

**Figure 7:**
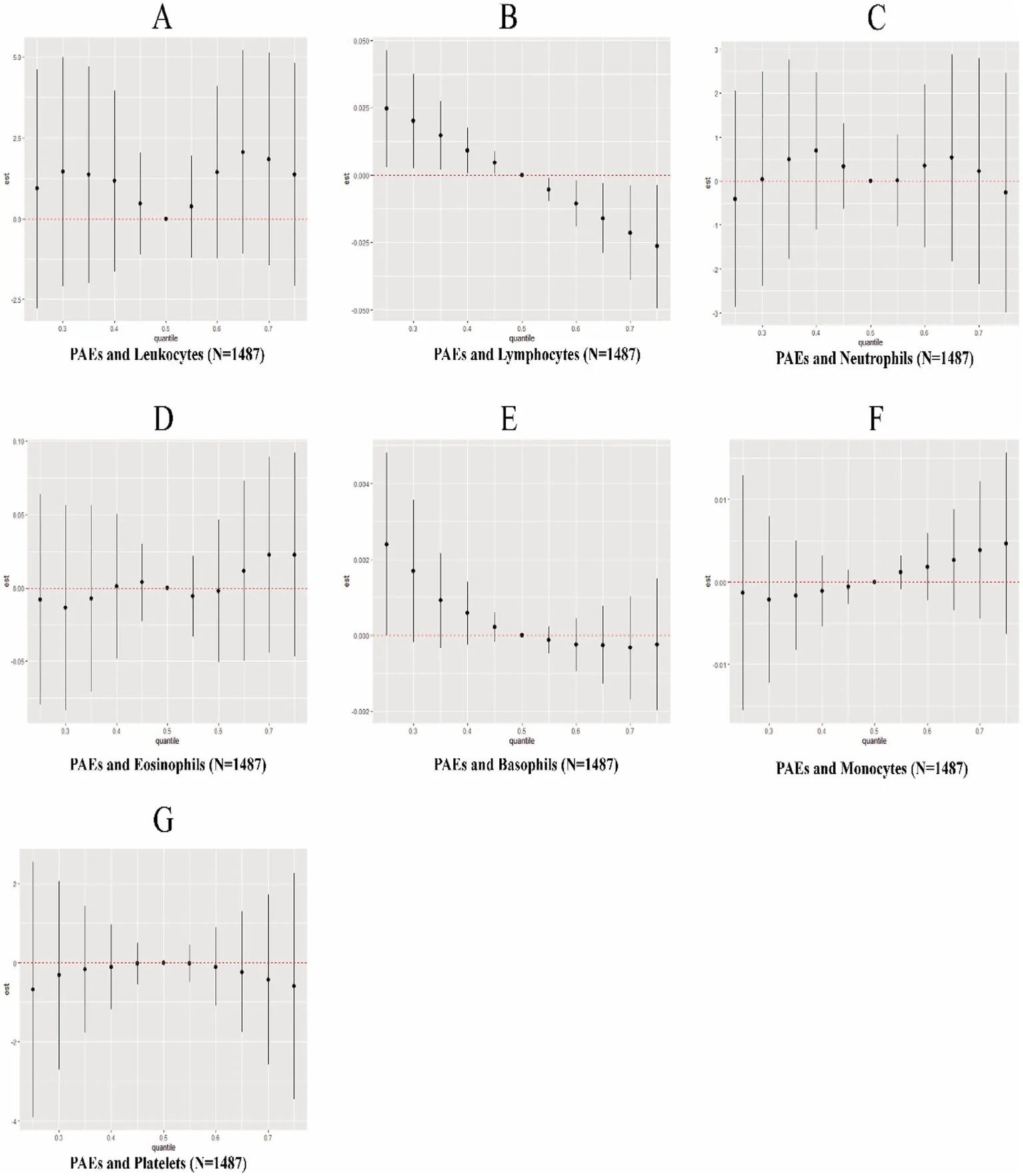
Combined associated between PAE metabolites and maternal immune cells in the third trimester. Adjustment factors: maternal age, maternal educational attainment, maternal occupation, maternal marital status, pre-pregnancy BMI, maternal smoking history, spousal educational attainment, spousal occupation, annual household income, gestational age and spousal smoking history. **(A)** Mixed association between PAE metabolites and leukocytes, **(B)** Mixed association between PAE metabolites and lymphocytes, **(C)** Mixed association between PAE metabolites and neutrophils, **(D)** Mixed association between PAE metabolites and eosinophils, **(E)** Mixed association between PAE metabolites and basophils, **(F)** Mixed association between PAE metabolites and monocytes, **(G)** Mixed association between PAE metabolites and platelets.

Previous studies have shown that PAE metabolites rarely occur in isolation; however, research has predominantly focused on the associated of individual pollutants rather than their combined impacts. To address this gap, we used Bayesian kernel machine regression (BKMR) to evaluate the combined associated between PAE metabolites and maternal immune cell profiles. In the first trimester, mixed exposure to PAE metabolites was significantly negatively correlated with white blood cell and neutrophil counts; no clear associations were observed with lymphocyte, eosinophil, basophil, monocyte, or platelet counts (Figure 5). In the second trimester, mixed PAE metabolite exposure showed negative correlations with lymphocyte and neutrophil levels, and positive correlations with eosinophil and basophil counts, though none of these associations were significant; no clear links were detected with white blood cell, monocyte, or platelet counts (Figure 6). In the third trimester, PAE metabolites were significantly negatively correlated with lymphocyte counts; they also exhibited a negative correlation with basophil counts and a positive correlation with monocyte counts (both non-significant), with no significant associations observed for white blood cell, neutrophil, eosinophil, or platelet counts (Figure 7).

## 4 Discussion

In this research, the urinary phthalate (PAE) concentrations of pregnant participants fluctuated between 0.08 and 124.33 μg/L creatinine (Cr). The exposure level was equivalent to the reported PAE range in Tianjin (0.803–122.025 μg/L Cr), while lower than the pollutant levels measured in Wuhan (14–206 μg/L Cr) and Hebei (9.4–253.9 μg/L Cr) (26–28). Additionally, the present cohort exhibited higher PAE accumulation than an Indian population (92.62–770 ng/L, converted as 0.09262–0.77 μg/L) (29). The geographical heterogeneity of PAE contamination may stem from regional differences in pollutant emission types, individual exposure loads, and unique environmental backgrounds in southwest China (1, 30). Consistent with prior evidence (22), maternal immune cell populations displayed obvious gestational variability. Specifically, white blood cells and neutrophils peaked in the third trimester; platelets and lymphocytes reached their minimum quantities in late pregnancy; eosinophils and basophils were most abundant during the second trimester. Nonetheless, such temporal variation differed from the outcomes recorded in an earlier study (23). Notably, this research merely compared immune indicators across trimesters and failed to capture continuous changes in immune cell abundance within a single gestational stage.

The current analysis demonstrated distinct trimester-dependent dose–response correlations and combined interactive associated between gestational PAE exposure and maternal immune profiles. These analytical outcomes were consistent with previous epidemiological evidence that PAE contamination triggers numerical changes in lymphocytes and monocytes (31, 32). The findings further validated the immunomodulatory properties of PAE contaminants (9, 33), which could suppress immune responses in both time-dependent and dose-dependent manners (34). Accumulated *in vivo* and *in vitro* data have collectively proven that PAE pollutants disrupt immune homeostasis (35–37). For example, even low-dose di(2-ethylhexyl) phthalate (DEHP) and dibutyl phthalate (DBP) can disturb intracellular immune signal transduction (38). Cross-species research further confirms that PAEs exert conservative immunosuppressive impacts on both invertebrates and vertebrates (39–41). Stage-stratified quantitative analysis indicated that mono(2-ethylhexyl) phthalate (MEHP) was solely correlated with monocyte counts in the second trimester, whereas mono(2-ethyl-5-carboxyhexyl) phthalate (MECPP) primarily affected neutrophil abundance in late gestation. This time-specific pattern was closely associated with the gestational modification of PAE metabolic enzyme activity driven by placental maturation (1). Although several studies have explored the correlation between PAEs and immune parameters (30, 31), few studies have systematically clarified such trimester-specific characteristics or distinguished the heterogeneous impacts of different exposure windows. Methodologically, Bayesian kernel machine regression (BKMR) was adopted in this study to evaluate the synergistic interactions among mixed PAE metabolites at diverse gestational stages. This approach effectively compensates for the defects of single-pollutant models and better simulates real-world human co-exposure conditions.

Although this study confirmed a statistical association between prenatal PAE exposure and alterations in the maternal circulatory immune cell profile, the exact molecular mechanisms underlying these pregnancy-specific, heterogeneous associations have not yet been fully elucidated. Therefore, this paper outlines three core mechanistic axes and further elaborates on them based on the findings of our empirical, stratified study. First, PAE metabolites (such as MEHP and MEOHP) disrupt intracellular signal transduction in various immune subsets, inhibit phagocytic function in monocytes and neutrophils, and induce excessive intracellular oxidative stress (42, 43). Existing studies have also identified MEHP-induced inflammatory cascades in monocytes. Second, PAEs disrupt the coordinated functioning of the maternal endocrine-immune axis by competitively binding to various nuclear receptors, including sex hormone receptors, thyroid hormone receptors, peroxisome proliferator-activated receptors (PPARs), and the aromatic hydrocarbon receptor (AhR) (44–46). The functional outcomes of these receptor-ligand interactions vary significantly depending on the pollutant subtype and stage of pregnancy: throughout pregnancy, dynamic changes in placental hormone synthesis and the activity of xenobiotic-metabolizing enzymes alter the bioavailability and intracellular accumulation of PAE metabolites. In late pregnancy, elevated levels of metabolic enzyme expression promote the biotransformation of maternal PAE into the active metabolites MEHP and MEOHP, which rapidly activate PPAR and AhR signaling pathways, thereby shifting the immune phenotype toward a pro-inflammatory or immunosuppressive state (47–49). Furthermore, PAE exposure regulates the transcription of immune-related genes, alters the catalytic activity of inflammatory and metabolic enzymes, promotes excessive production of reactive oxygen species (ROS) within cells, inhibits classical immune signaling cascades, and interferes with complement system activation (50). The interactions among oxidative stress, nuclear receptor signaling, and the regulation of inflammatory genes form a synergistic toxic network, the overall intensity of which depends on pregnancy-specific placental metabolic capacity (51). The increased burden of active PAE metabolites during the third trimester reinforces these synergistic pathways, ultimately disrupting maternal immune homeostasis and generating the pregnancy-stratified heterogeneous associations we identified in our RCS and regression analyses (52).

## 5 Strengths and limitations

This study possesses several methodological strengths. We integrated multiple statistical frameworks to quantify trimester-specific associations between PAE exposure and maternal circulating immune profiles, alongside characterization of their potential non-linear dose–response relationships. Nevertheless, the present work has notable limitations that warrant consideration. First, the cross-sectional study design precludes causal inference linking PAE exposure to maternal immune dysregulation. This framework cannot capture longitudinal shifts in urinary PAE metabolites and peripheral immune cell populations across gestation; future prospective cohorts with serial biological specimen collection are required to strengthen causal interpretability. Second, pregnant individuals are ubiquitously co-exposed to a suite of environmental contaminants, including heavy metals and alternative endocrine-disrupting compounds, which may exert interactive immunomodulatory associated alongside PAEs. We did not account for these co-occurring confounding exposures, which may mask uncharacterized pollutant interactions and diminish the generalizability of our population-based findings. Third, urine specimens were collected using plastic centrifugation tubes, which risk exogenous PAE leaching and introduce quantification bias for metabolite measurements. Rigorous quality-control protocols were implemented to mitigate this contamination risk, yet this experimental artifact remains an unavoidable constraint. Fourth, this epidemiological investigation lacks mechanistic interrogation. The molecular regulatory networks, genetic pathways and intracellular signaling cascades underpinning PAE-mediated immune perturbation remain unelucidated. Follow-up mechanistic research is essential to furnish molecular insights and inform targeted intervention strategies for PAE-associated immune dysregulation in pregnant cohorts.

## 6 Conclusion

In this study, we found that PAE metabolites in the urine of pregnant women were correlated with maternal immune cell counts at various stages of pregnancy, highlighting the potential immunomodulatory effects of PAE exposure during pregnancy. However, since this study is primarily based on epidemiological data, all findings should be interpreted with caution.

## Data Availability

The original contributions presented in the study are included in the article/Supplementary material, further inquiries can be directed to the corresponding author/s.
