# Supplementary material for: Associations between prenatal phthalate exposure and maternal immune cell profiles across trimesters
Source: Front Public Health. 2026 Sep 1;14:1912015. doi: 10.3389/fpubh.2026.1912015 (PMC13577178; doi:10.3389/fpubh.2026.1912015)
Supplement: Supplementary file 1 [file Supplementary_file_1.docx]

| **Table S1** On-board specific information table for phthalate metabolites | | | | |
| --- | --- | --- | --- | --- |
| metabolite | retention time | parent ion | daughter ion | collision energy |
| MMP | 16.731 | 237 | 89 | 15 |
| MEP | 17.837 | 251 | 75 | 15 |
| MIBP | 17.840 | 223 | 75 | 15 |
| MBP | 19.785 | 223 | 75 | 15 |
| MOP | 20.426 | 223 | 73 | 15 |
| MEHP | 21.669 | 221 | 73 | 15 |
| MEOHP | 24.633 | 221 | 73 | 15 |
| MBZP | 27.261 | 179 | 105 | 20 |
| MEHHP | 29.367 | 221 | 73 | 15 |
| MECPP | 31.667 | 221 | 73 | 15 |

**Table S2** Limit of detection (LOD), limit of quantification (LOQ), recovery rate, and precision.

| compound | internal standard | regression equation | Decision coefficient (R^2^) | LOD (ug/L) | LOQ (ug/L) | recovery rate（R%） | precision（%） |
| --- | --- | --- | --- | --- | --- | --- | --- |
| MMP | MEHP-C4 | y=0.0037x+5.9620 | 0.9986 | 0.0375 | 0.1250 | 80.45 | 8.37 |
| MEP | MEHP-C4 | Y=0.9972x+0.0202 | 0.9972 | 0.0288 | 0.0962 | 102.31 | 6.15 |
| MIBP | MEHP-C4 | Y=0.1002x+0.0813 | 0.9988 | 0.0023 | 0.0075 | 99.71 | 4.95 |
| MBP | MEHP-C4 | Y=0.1681x+0.1936 | 0.9982 | 0.0015 | 0.0051 | 92.64 | 4.77 |
| MOP | MEHP-C4 | Y=0.1300x+0.0021 | 0.9997 | 0.0625 | 0.2083 | 100.00 | 3.15 |
| MEHP | MEHP-C4 | Y=0.1090x+0.0234 | 0.9999 | 0.0326 | 0.1087 | 112.59 | 3.59 |
| MEOHP | MEHHP-C4 | Y=0.0834x-0.0876 | 0.9986 | 1.0714 | 3.5714 | 105.16 | 4.14 |
| MBZP | MEHP-C4 | Y=0.0877x+0.0351 | 0.9976 | 0.0405 | 0.1351 | 92.01 | 5.83 |
| MEHHP | MEHHP-C4 | y=0.2029x-0.0621 | 0.9998 | 0.0103 | 0.1087 | 104.21 | 3.67 |
| MECPP | MEHHP-C4 | Y=0.0061x-0.0070 | 0.9847 | 23.4375 | 37.125 | 134.28 | 54.40 |

| **Table S3** Maternal urinary concentrations of PAE metabolites and blood levels of immune cells | | | | | |
| --- | --- | --- | --- | --- | --- |
|  | The First Trimester  50^th^ (25^th^,75^th^) | The Second Trimester  50^th^ (25^th^,75^th^) | The Third Trimester  50^th^ (25^th^,75^th^) | *F* | *P* |
| Leukocytes (×10⁹/L) | 7.20 (8.20, 9.50) | 8.10 (9.49, 10.92) | 8.18 (9.70, 11.60) | 49.12 | **<.001** |
| Lymphocytes(×10⁹/L) | 1.48 (1.77, 2.14) | 1.44 (1.73, 2.04) | 1.31 (1.62, 1.93) | 28.99 | **<.001** |
| Neutrophils (×10⁹/L) | 4.89 (5.92, 6.98) | 5.96 (7.18, 8.46) | 5.97 (7.40, 9.20) | 64.27 | **<.001** |
| Eosinophils (×10⁹/L) | 0.04 (0.08, 0.12) | 0.05 (0.08, 0.13) | 0.03 (0.06, 0.10) | 49.27 | **<.001** |
| Basophils (×10⁹/L) | 0.01 (0.02, 0.03) | 0.02 (0.02, 0.03) | 0.01 (0.02, 0.02) | 95.92 | **<.001** |
| Monocytes (×10⁹/L) | 0.30(0.37, 0.45) | 0.36 (0.45, 0.55) | 0.42 (0.52, 0.65) | 150.11 | **<.001** |
| Platelets (×10⁹/L) | 204.0(244.0,283.0) | 188.0(223.0, 258.0) | 170.0(204.0, 245.0) | 55.49 | **<.001** |
| MMP (μg/L Cr) | 2.54 (0.20, 6.85) | 1.85(0.10, 8.19) | 2.43(0.15, 8.62) | 0.77 | 0.464 |
| MEP (μg/L Cr) | 11.63(3.80, 29.56) | 12.01(3.75, 28.23) | 11.55(3.73, 29.47) | 3.45 | **0.032** |
| MIBP (μg/L Cr) | 40.13(18.48, 89.04) | 40.34 (17.03, 89.82) | 33.91(14.14, 75.72) | 4.42 | **0.012** |
| MBP (μg/L Cr) | 113.44 (47.44, 259.26) | 124.33(48.14,292.61) | 112.57(45.13, 265.60) | 1.87 | 0.154 |
| MEHP (μg/L Cr) | 15.87(2.95, 64.35) | 13.76(3.51, 79.74) | 16.55(4.01, 65.20) | 3.14 | **0.044** |
| MOP (μg/L Cr) | 0.16(0.08, 0.42) | 0.17(0.07, 0.39) | 0.15(0.07, 0.37) | 0.31 | 0.732 |
| MBZP (μg/L Cr) | 0.10 (0.05, 0.34) | 0.09 (0.04, 0.27) | 0.09(0.04, 0.27) | 0.96 | 0.384 |
| MEOHP (μg/L Cr) | 8.74 (3.60, 21.28) | 8.19(2.86, 23.65) | 7.96(3.05, 24.44) | 5.27 | **0.005** |
| MEHHP (μg/L Cr) | 9.62(3.88, 20.73) | 9.21 (3.58, 23.23) | 8.53 (2.78, 21.05) | 4.29 | **0.014** |
| MECPP (μg/L Cr) | 79.74(30.80, 279.23) | 77.92(31.53, 258.88) | 73.50(29.51, 214.07) | 3.19 | **0.041** |

| **Table S4** Association between PAE metabolites and leukocytes and eosinophils | | | | | | |
| --- | --- | --- | --- | --- | --- | --- |
|  |  | leukocytes |  |  | eosinophils |  |
|  | The First Trimester  β (95CI%) | The Second Trimester  β (95CI%) | The Third Trimester  β (95CI%) | The First Trimester  β (95CI%) | The Second Trimester  β (95CI%) | The Third Trimester  β (95CI%) |
| MMP | -0.196(-0.484, 0.092) | -0.109(-0.285, 0.068) | 0.155(0.010, 0.299) | -0.002(-0.071, 0.067) | -0.035(-0.071, 0.001) | -0.005(-0.032, 0.022) |
| MEP | -0.235(-0.581, 0.112) | 0.051(-0.157, 0.258) | 0.012(-0.162, 0.187) | -0.046(-0.129, 0.037) | 0.015(-0.027, 0.057) | -0.017(-0.049, 0.016) |
| MIBP | 0.052(-0.479, 0.584) | 0.079(-0.241, 0.399) | -0.151(-0.355, 0.053) | 0.004(-0.123, 0.131) | 0.051(-0.014, 0.117) | 0.027(-0.011, 0.065) |
| MBP | 0.416(-0.216, 1.049) | 0.132(-0.241, 0.504) | -0.159(-0.437, 0.118) | 0.019(-0.132, 0.171) | -0.020(-0.096, 0.056) | -0.022(-0.074, 0.030) |
| MEHP | 0.121(-0.138, 0.380) | -0.141(-0.309, 0.027) | 0.164(0.019, 0.309) | 0.038(-0.024, 0.100) | -0.042(-0.076, -0.008) | -0.022(-0.049, 0.005) |
| MOP | -0.408(-0.891, 0.076) | 0.338(0.006, 0.670) | -0.151(-0.411, 0.110) | -0.023(-0.138, 0.093) | 0.070(0.003, 0.138) | -0.009(-0.057, 0.040) |
| MBZP | 0.133(-0.312, 0.577) | -0.103(-0.419, 0.213) | -0.014(-0.255, 0.226) | 0.069(-0.038, 0.175) | -0.025(-0.089, 0.040) | 0.009(-0.036, 0.054) |
| MEOHP | -0.399(-0.838, 0.041) | 0.125(-0.163, 0.414) | 0.099(-0.122, 0.320) | -0.011(-0.116, 0.094) | 0.042(-0.017, 0.100) | -0.035(-0.076, 0.006) |
| MEHHP | -0.102(-0.373, 0.168) | 0.016(-0.151, 0.184) | -0.003(-0.136, 0.131) | -0.004(-0.068, 0.061) | 0.007(-0.027, 0.041) | 0.017(-0.008, 0.041) |
| MECPP | -0.011(-0.363, 0.342) | 0.024(-0.221, 0.268) | -0.053(-0.266, 0.161) | -0.032(-0.116, 0.052) | -0.053(-0.102, -0.003) | 0.007(-0.033, 0.046) |
| Adjustment factors: maternal age, maternal educational attainment, maternal occupation, maternal marital status, pre-pregnancy BMI, maternal smoking history, spousal educational attainment, spousal occupation, annual household income, gestational age and spousal smoking history | | | | | | |

| **Table S5** Association between PAE metabolites and neutrophils and eosinophils | | | | | | |
| --- | --- | --- | --- | --- | --- | --- |
|  |  | neutrophils |  |  | eosinophils |  |
|  | The First Trimester  β (95CI%) | The Second Trimester  β (95CI%) | The Third Trimester  β (95CI%) | The First Trimester  β (95CI%) | The Second Trimester  β (95CI%) | The Third Trimester  β (95CI%) |
| MMP | -0.202(-0.459, 0.056) | -0.064(-0.219, 0.091) | 0.154(0.021, 0.287) | 0.000(-0.013, 0.014) | -0.002(-0.009, 0.006) | 0.001(-0.003, 0.005) |
| MEP | -0.191(-0.501, 0.119) | 0.029(-0.154, 0.211) | 0.033(-0.127, 0.194) | 0.013(-0.003, 0.029) | -0.001(-0.010, 0.008) | -0.001(-0.006, 0.004) |
| MIBP | 0.052(-0.423, 0.527) | 0.026(-0.255, 0.308) | -0.166(-0.354, 0.021) | -0.014(-0.039, 0.010) | 0.002(-0.012, 0.016) | 0.000(-0.006, 0.006) |
| MBP | 0.406(-0.160, 0.971) | 0.147(-0.181, 0.474) | -0.139(-0.394, 0.117) | 0.008(-0.021, 0.038) | -0.005(-0.021, 0.011) | 0.006(-0.002, 0.014) |
| MEHP | 0.058(-0.174, 0.290) | -0.086(-0.234, 0.061) | 0.180(0.046, 0.313) | 0.010(-0.002, 0.022) | -0.001(-0.008, 0.006) | -0.006(-0.010, -0.002) |
| MOP | -0.362(-0.794, 0.071) | 0.257(-0.035, 0.548) | -0.120(-0.359, 0.120) | -0.007(-0.030, 0.015) | -0.002(-0.017, 0.012) | 0.000(-0.007, 0.008) |
| MBZP | 0.064(-0.334, 0.461) | -0.088(-0.366, 0.189) | -0.027(-0.248, 0.195) | 0.003(-0.018, 0.023) | 0.005(-0.009, 0.018) | 0.002(-0.005, 0.009) |
| MEOHP | -0.363(-0.756, 0.030) | 0.094(-0.160, 0.348) | 0.124(-0.078, 0.327) | -0.014(-0.034, 0.007) | -0.002(-0.014, 0.011) | 0.000(-0.006, 0.007) |
| MEHHP | -0.098(-0.340, 0.143) | -0.004(-0.151, 0.143) | -0.020(-0.143, 0.102) | -0.005(-0.017, 0.008) | 0.005(-0.002, 0.012) | 0.001(-0.003, 0.005) |
| MECPP | 0.022(-0.293, 0.337) | 0.082(-0.133, 0.296) | -0.057(-0.253, 0.139) | 0.002(-0.014, 0.019) | -0.003(-0.013, 0.008) | -0.004(-0.010, 0.003) |
| Adjustment factors: maternal age, maternal educational attainment, maternal occupation, maternal marital status, pre-pregnancy BMI, maternal smoking history, spousal educational attainment, spousal occupation, annual household income, gestational age and spousal smoking history | | | | | | |

| **Table S6** Association between PAE metabolites and eosinophils and monocytes | | | | | | |
| --- | --- | --- | --- | --- | --- | --- |
|  |  | eosinophils |  |  | monocytes |  |
|  | The First Trimester  β (95CI%) | The Second Trimester  β (95CI%) | The Third Trimester  β (95CI%) | The First Trimester  β (95CI%) | The Second Trimester  β (95CI%) | The Third Trimester  β (95CI%) |
| MMP | 0.001(0.000, 0.003) | -0.001(-0.002, 0.000) | -0.001(-0.002, 0.000) | 0.007(-0.010, 0.024) | -0.007(-0.019, 0.005) | 0.006(-0.005, 0.017) |
| MEP | -0.001(-0.003, 0.001) | 0.000(-0.001, 0.002) | 0.000(-0.001, 0.001) | -0.011(-0.031, 0.009) | 0.007(-0.006, 0.021) | -0.001(-0.014, 0.012) |
| MIBP | -0.002(-0.005, 0.001) | 0.001(-0.001, 0.003) | 0.002(0.001, 0.003) | 0.008(-0.023, 0.039) | -0.003(-0.025, 0.018) | -0.018(-0.033, -0.002) |
| MBP | 0.002(-0.001, 0.006) | 0.001(-0.002, 0.003) | 0.000(-0.002, 0.001) | -0.015(-0.052, 0.021) | 0.011(-0.014, 0.036) | -0.006(-0.027, 0.016) |
| MEHP | 0.000(-0.001, 0.002) | 0.000(-0.001, 0.001) | 0.000(-0.001, 0.000) | 0.014(-0.001, 0.029) | -0.010(-0.021, 0.001) | 0.011(0.000, 0.022) |
| MOP | 0.000(-0.003, 0.003) | 0.002(0.000, 0.003) | 0.001(0.000, 0.003) | -0.016(-0.044, 0.013) | 0.010(-0.012, 0.032) | -0.023(-0.043, -0.003) |
| MBZP | -0.002(-0.004, 0.001) | -0.001(-0.003, 0.001) | -0.002(-0.003, 0.000) | 0.005(-0.020, 0.031) | 0.009(-0.012, 0.030) | 0.002(-0.017, 0.020) |
| MEOHP | -0.002(-0.004, 0.001) | 0.001(-0.001, 0.002) | -0.001(-0.002, 0.000) | -0.010(-0.036, 0.015) | -0.009(-0.028, 0.011) | 0.008(-0.009, 0.025) |
| MEHHP | 0.001(0.000, 0.003) | 0.000(-0.001, 0.001) | 0.000(-0.001, 0.001) | 0.003(-0.013, 0.019) | 0.008(-0.003, 0.019) | 0.001(-0.009, 0.011) |
| MECPP | 0.000(-0.002, 0.002) | 0.000(-0.002, 0.001) | 0.001(-0.001, 0.002) | -0.003(-0.023, 0.017) | -0.003(-0.020, 0.013) | 0.001(-0.016, 0.017) |
| Adjustment factors: maternal age, maternal educational attainment, maternal occupation, maternal marital status, pre-pregnancy BMI, maternal smoking history, spousal educational attainment, spousal occupation, annual household income, gestational age and spousal smoking history | | | | | | |

| **Table S7** Association of PAE metabolites and platelets | | | |
| --- | --- | --- | --- |
|  | The First Trimester  β (95CI%) | The Second Trimester  β (95CI%) | The Third Trimester  β (95CI%) |
| MMP | -2.672(-10.945, 5.601) | -2.383(-6.697, 1.931) | 1.086(-2.348, 4.520) |
| MEP | 5.632(-4.310, 15.574) | 1.402(-3.670, 6.475) | 1.308(-2.840, 5.456) |
| MIBP | -7.826(-23.080, 7.428) | -3.528(-11.356, 4.301) | 3.566(-1.285, 8.417) |
| MBP | 4.799(-13.350, 22.949) | 3.586(-5.516, 12.687) | -1.442(-8.040, 5.156) |
| MEHP | 1.335(-6.107, 8.777) | 2.158(-1.946, 6.263) | 3.391(-0.062, 6.843) |
| MOP | -4.043(-17.925, 9.839) | 0.158(-7.961, 8.277) | 0.520(-5.668, 6.707) |
| MBZP | 1.437(-11.332, 14.206) | 0.046(-7.679, 7.771) | -2.639(-8.358, 3.080) |
| MEOHP | -3.004(-15.621, 9.614) | 0.279(-6.785, 7.344) | -5.575(-10.817, -0.334) |
| MEHHP | -1.746(-9.501, 6.010) | 1.772(-2.324, 5.868) | 2.301(-0.868, 5.470) |
| MECPP | 1.050(-9.060, 11.160) | -4.161(-10.131, 1.810) | -2.022(-7.081, 3.037) |
| Adjustment factors: maternal age, maternal educational attainment, maternal occupation, maternal marital status, pre-pregnancy BMI, maternal smoking history, spousal educational attainment, spousal occupation, annual household income, gestational age and spousal smoking history | | | |

| **Table S8** Posterior Inclusion Probability (PIP) Between PAE Metabolites and Immune Cells in the first trimester | | | | | | | |
| --- | --- | --- | --- | --- | --- | --- | --- |
|  | leukocytes | Lymphocytes | Neutrophils | Eosinophils | Basophils | Monocytes | Platelets |
| MMP | 0.066 | 0.000 | 0.146 | 0.101 | 0.030 | 0.009 | 0.019 |
| MEP | 0.231 | 0.027 | 0.240 | 0.150 | 0.009 | 0.098 | 0.013 |
| MIBP | 0.005 | 0.006 | 0.142 | 0.168 | 0.008 | 0.032 | 0.067 |
| MBP | 0.074 | 0.006 | 0.270 | 0.148 | 0.065 | 0.023 | 0.014 |
| MEHP | 0.028 | 0.044 | 0.105 | 0.285 | 0.034 | 0.016 | 0.041 |
| MOP | 0.109 | 0.043 | 0.236 | 0.143 | 0.060 | 0.041 | 0.110 |
| MBZP | 0.046 | 0.037 | 0.253 | 0.151 | 0.216 | 0.030 | 0.046 |
| MEOHP | 0.359 | 0.005 | 0.400 | 0.181 | 0.045 | 0.065 | 0.102 |
| MEHHP | 0.020 | 0.031 | 0.160 | 0.108 | 0.012 | 0.015 | 0.032 |
| MECPP | 0.076 | 0.073 | 0.135 | 0.122 | 0.003 | 0.056 | 0.032 |
| Adjustment factors: maternal age, maternal educational attainment, maternal occupation, maternal marital status, pre-pregnancy BMI, maternal smoking history, spousal educational attainment, spousal occupation, annual household income, gestational age and spousal smoking history | | | | | | | |

| **Table S9** Posterior Inclusion Probability (PIP) Between PAE Metabolites and Immune Cells in the second trimester | | | | | | | |
| --- | --- | --- | --- | --- | --- | --- | --- |
|  | leukocytes | Lymphocytes | Neutrophils | Eosinophils | Basophils | Monocytes | Platelets |
| MMP | 1.000 | 0.128 | 0.330 | 0.618 | 0.03 | 0.315 | 0.887 |
| MEP | 1.000 | 0.117 | 0.338 | 0.605 | 0.010 | 0.339 | 0.792 |
| MIBP | 0.898 | 0.267 | 0.336 | 0.661 | 0.106 | 0.309 | 0.824 |
| MBP | 1.000 | 0.152 | 0.338 | 0.688 | 0.026 | 0.323 | 0.804 |
| MEHP | 1.000 | 0.211 | 0.330 | 0.574 | 0.001 | 0.332 | 0.882 |
| MOP | 1.000 | 0.159 | 0.364 | 0.598 | 0.056 | 0.333 | 0.879 |
| MBZP | 0.980 | 0.122 | 0.338 | 0.589 | 0.078 | 0.404 | 0.817 |
| MEOHP | 0.875 | 0.116 | 0.324 | 0.551 | 0.000 | 0.316 | 0.871 |
| MEHHP | 1.000 | 0.124 | 0.354 | 0.618 | 0.003 | 0.311 | 0.864 |
| MECPP | 0.992 | 0.166 | 0.329 | 0.533 | 0.035 | 0.355 | 0.873 |
| Adjustment factors: maternal age, maternal educational attainment, maternal occupation, maternal marital status, pre-pregnancy BMI, maternal smoking history, spousal educational attainment, spousal occupation, annual household income, gestational age and spousal smoking history | | | | | | | |

| **Table S10** Posterior Inclusion Probability (PIP) Between PAE Metabolites and Immune Cells in the third trimester | | | | | | | |
| --- | --- | --- | --- | --- | --- | --- | --- |
|  | leukocytes | Lymphocytes | Neutrophils | Eosinophils | Basophils | Monocytes | Platelets |
| MMP | 1.000 | 0.000 | 0.854 | 0.977 | 0.660 | 0.062 | 0.003 |
| MEP | 1.000 | 0.000 | 1.000 | 1.000 | 0.548 | 0.251 | 0.088 |
| MIBP | 0.974 | 0.000 | 0.923 | 0.840 | 1.000 | 0.537 | 0.110 |
| MBP | 0.966 | 0.012 | 0.920 | 0.876 | 0.382 | 0.040 | 0.058 |
| MEHP | 1.000 | 0.053 | 1.000 | 1.000 | 0.242 | 0.081 | 0.000 |
| MOP | 1.000 | 0.000 | 0.952 | 0.983 | 0.859 | 0.099 | 0.020 |
| MBZP | 1.000 | 0.043 | 1.000 | 0.902 | 0.702 | 0.144 | 0.011 |
| MEOHP | 1.000 | 0.096 | 1.000 | 0.912 | 0.702 | 0.169 | 0.023 |
| MEHHP | 0.984 | 0.000 | 0.960 | 0.871 | 0.316 | 0.000 | 0.000 |
| MECPP | 1.000 | 0.038 | 1.000 | 1.000 | 0.314 | 0.020 | 0.031 |
| Adjustment factors: maternal age, maternal educational attainment, maternal occupation, maternal marital status, pre-pregnancy BMI, maternal smoking history, spousal educational attainment, spousal occupation, annual household income, gestational age and spousal smoking history | | | | | | | |
