# Supplementary material for: Associations between prenatal phthalate exposure and maternal immune cell profiles across trimesters
Source: Front Public Health. 2026 Sep 1;14:1912015. doi: 10.3389/fpubh.2026.1912015 (PMC13577178; doi:10.3389/fpubh.2026.1912015)
Supplement: Supplementary file 2 [file Supplementary_file_2.docx]

ZBC(**N=6583**)

Complete survey data **(N=6359)**：including maternal age, race, education, occupation, marital status, pre-pregnancy body mass index, smoking history, number of pregnancies, spousal education, spousal occupation, annual household income, and spousal smoking history.

Maternal immunocyte **(N=3461)**: leukocytes, lymphocytes, neutrophils, eosinophils, basophils, monocytes, and platelets.

Data on the detection of PAEs in urine during pregnancy **(N=2581)**：including: Monoethyl phthalate (MEP), monobutyl phthalate (MBP), monobutyl phthalate (MiBP) and monomethyl phthalate (MMP)), monobutyl phthalate (MnBP), mono(2-ethylhexyl) phthalate (MEHP), mono(2-ethyl-5-hydroxyhexyl) phthalate (MEHHP), mono(2-ethyl-5-oxohexyl) phthalate (MEOHP), mono(2-ethyl-5) octyl phthalate (MOP), monophenyl phthalate (MBzP)

Study population **(N=2581**): The sample sizes were 287 in early pregnancy, 807 in mid-pregnancy, and 1,487 in late pregnancy.

The sample sizes were 287 in early pregnancy, 807 in mid-pregnancy, and 1,487 in late pregnancy.

Figure S1 Study population

Figure S2: Correlation Analysis between Phthalate Metabolites. All metabolites, except MMP, MBZP, and MECPP, displayed strong correlation with each other. All metabolites, it should be noted that stronger correlations are represented by larger shaded circles and darker colors numbers, although all correlations are relatively weak.

Figure S3 Association between PAE metabolites and leukocytes in the first trimester

Figure S4 Association between PAE metabolites and Lymphocytes in the first trimester

Figure S5 Association between PAE metabolites and Neutrophils in the first trimester

Figure S6 Association between PAE metabolites and Eosinophils in the first trimester

Figure S7 Association between PAE metabolites and Basophils in the first trimester

Figure S8Association between PAE metabolites and Monocytes in the first trimester

Figure S9Association between PAE metabolites and Platelets in the first trimester

Figure S10 Association between PAE metabolites and leukocytes in the second trimester

Figure S11 Association between PAE metabolites and Lymphocytes in the second trimester

Figure S12 Association between PAE metabolites and Neutrophils in the second trimester

Figure S13 Association between PAE metabolites and Eosinophils in the second trimester

Figure S14 Association between PAE metabolites and Basophils in the second trimester

Figure S15Association between PAE metabolites and Monocytes in the second trimester

Figure S16Association between PAE metabolites and Platelets in the second trimester

Figure S17 Association between PAE metabolites and leukocytes in the third trimester

Figure S18 Association between PAE metabolites and Lymphocytes in the third trimester

Figure S19 Association between PAE metabolites and Neutrophils in the third trimester

Figure S20 Association between PAE metabolites and Eosinophils in the third trimester

Figure S21 Association between PAE metabolites and Basophils in the third trimester

Figure S22Association between PAE metabolites and Monocytes in the third trimester

Figure S23Association between PAE metabolites and Platelets in the third trimester
